# Trop2 and its overexpression in cancers: regulation and clinical/therapeutic implications

**DOI:** 10.18632/genesandcancer.40

**Published:** 2015-03

**Authors:** Anna Shvartsur, Benjamin Bonavida

**Affiliations:** ^1^ Department of Microbiology, Immunology and Molecular Genetics, Jonsson Comprehensive Cancer Center and David Geffen School of Medicine, University of California at Los Angeles, Los Angeles, CA, USA

## Abstract

Trop2 is a transmembrane glycoprotein encoded by the *Tacstd2* gene. It is an intracellular calcium signal transducer that is differentially expressed in many cancers. It signals cells for self-renewal, proliferation, invasion, and survival. It has stem cell-like qualities. Trop2 is expressed in many normal tissues, though in contrast, it is overexpressed in many cancers and the overexpression of Trop2 is of prognostic significance. Several ligands have been proposed that interact with Trop2. Trop2 signals the cells via different pathways and it is transcriptionally regulated by a complex network of several transcription factors. Trop2 expression in cancer cells has been correlated with drug resistance. Several strategies target Trop2 on cancer cells that include antibodies, antibody fusion proteins, chemical inhibitors, nanoparticles, etc. The *in vitro* studies and pre-clinical studies, using these various therapeutic treatments, have resulted in significant inhibition of tumor cell growth both *in vitro* and *in vivo* in mice. A clinical study is underway using IMMU-132 (hrS7 linked to SN38) in patients with epithelial cancers. This review describes briefly the various characteristics of cancer cells overexpressing Trop2 and the potential application of Trop2 as both a prognostic biomarker and as a therapeutic target to reverse resistance.

## INTRODUCTION

The transmembrane glycoprotein Trop2 is highly expressed in many cancers, but not all, and has differential expression in certain normal tissues. Trop2 is also known as trophoblast antigen 2, cell surface glycoprotein Trop-2/Trop2, gastrointestinal tumor-associated antigen GA7331, pancreatic carcinoma marker protein GA733-1/GA733, membrane component chromosome 1 surface marker 1 M1S1, epithelial glycoprotein-1, EGP-1, CAA1, Gelatinous Drop-Like Corneal Dystrophy GDLD, and TTD2 [1,2]. It is coded by the gene *Tacstd2.* It is about 35 kDa [3]. Trop2 spans the cellular membrane: it has an extracellular, a transmembrane, and an intracellular domain, along with a cytoplasmic tail essential for signaling [4].

Trop2 was first discovered in trophoblast cells. Trophoblast cells possess the ability to invade uterine decidua during placental implantation. Lipinski et al, [5] raised monoclonal antibodies against human neoplastic choriocarcinoma trophoblast cell lines via hybridoma technology. This led to the discovery of four new protein antigens (Trop1, 2, 3, and 4) expressed on normal and malignant trophoblast cells. Trop2 was reported to be expressed on syncytio- and cytotrophoblasts [5]. Trop2 may analogously confer the capacity for proliferation and invasion to cancer cells [2,6]. Trop2 is expressed in the cytoplasm when cells become malignant and in some cases of cancer metastasis and recurrence [7].

Trop2 has been implicated in numerous intracellular signaling pathways. Trop2 transduces an intracellular calcium signal. Trop2-induced signal transduction can occur without extracellular Ca^2+^, suggesting a mobilization of Ca^2+^ from internal stores. Specific antibodies are used for cross-linking Trop2. This cross-linking leads to a significant rise in cytoplasmic Ca^2+^ [4]. Trop2 provides crucial signals for cells with requirements for proliferation, survival, self-renewal, and invasion [8]. Trop2 has several ligands, inlcluding claudin-1, claudin-7, cyclin D1, and potentially IGF-1. Trop2 has stem cell-like qualities and regulates cell growth, transformation, regeneration, and proliferation, which explains why its overexpression can lead to tumor progression. It is expressed on the surface of many stem/progenitor cells and has a role in maintaining tight junction integrity [9].

Trop2 might be a modulator and/or an enhancer of EpCAM-induced cell signaling. Trop2 modulation of EpCAM can cause EpCAM to proliferate and migrate into liver parenchyma [4]. Trop2 can foster cell migration without the presence of growth factors. Induced foci formation represents a loss of the ability to maintain cell growth and movement [8].

Regulated Intramembrane Proteolysis (RIP) is required for Trop2 activity; it is necessary for Trop2's enhanced cell growth and self-renewal activity in prostate cancer. RIP cleaves Trop2 through the TNF-α converting enzyme (TACE) followed by γ-secretase cleavage within the transmembrane domain. Cleavage is mediated by presenilin 1 (PS-1), which is the dominant enzyme, and presenilin 2 (PS-2). This cleavage makes two products, namely the extracellular domain (ECD) and the intracellular domain (ICD) [10].

The ECD is shed and found only on the plasma membrane and in the cytoplasm. Secreted ECD causes an increase in sphere size but not in sphere number, which suggests that the ECD increases the proliferation of progenitor cells, specifically of prostate stem cells. Treating prostate cells with secreted ECD leads to the appearance of small 6 kD fragments, suggesting Trop2 cleavage. It is uncertain whether the ECD induces Trop2 cleavage via distinct binding partner interactions or through direct hydrophilic interactions [10].

**Figure 1:**
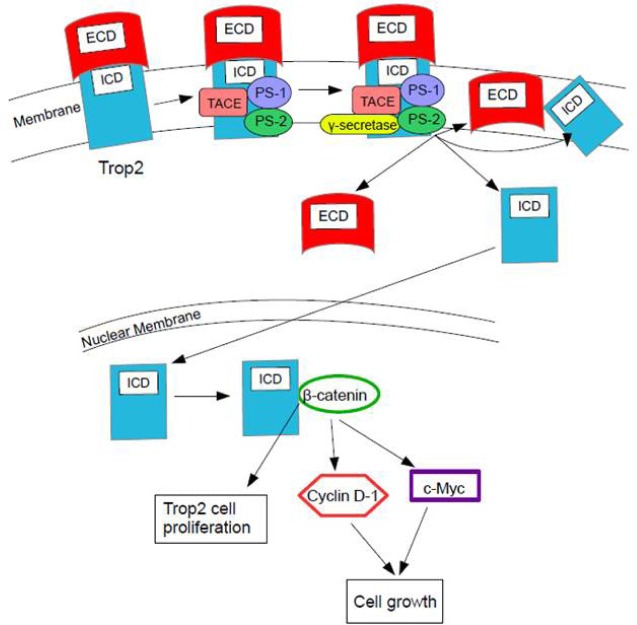
Trop2-Regulated Intramembrane Proteolysis (RIP) RIP is required for Trop2 activity in prostate cancer. Trop2 is cleaved by the TNF-α converting enzyme (TACE), followed by y-secretase. The cleavage is mediated by the enzymes PS-1 and PS-2 in the complex. PS-1 is the dominant enzyme. Two products are made: the extracellular domain (ECD) is shed and is found on the plasma membrane and in the cytoplasm, and the intracellular domain (ICD) is released from the membrane and accumulates in the nucleus (although some is found on the membrane). The ICD is the functionally dominant part of Trop2 in prostate cancer. Within prostate cancer regions, β-catenin colocalizes with the ICD in the nucleus, leading to Trop2 proliferation. This process could possibly occur in other cancers. This colocalization causes upregulation of the downstream targets cyclin D1 and c-myc, which leads to cell growth [10].

The ICD is released from the membrane, for the most part, and accumulates in the nucleus. Nuclear ICD is only detected in cancer specimens. Cleavage and activation is required for its transformation activity and it has been associated with human prostate cancer, but it could also be associated with other cancers [10]. The ICD is the functionally dominant part of Trop2. It promotes self-renewal, initiates prostatic intraepithelial neoplasia (PIN) and is involved in a β-catenin-dependent signaling cascade. Figure 1 shows the process of RIP activity and the interaction of the ICD with β-catenin [8].

**Figure 2:**
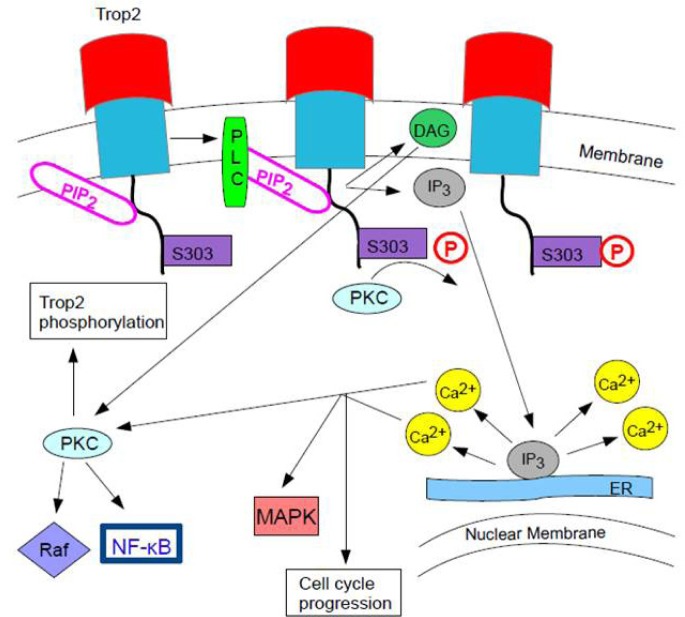
PLC Cleavage of PIP2 via Trop2 S303 Phosphorylation If the cytoplasmic tail of Trop2 is bound to PIP_2_, it might be concentrating Trop2 for hydrolysis by phospholipase C (PLC). Once position S_303_ on the Trop2's cytoplasmic tail is phosphorylated by protein kinase C (PKC), PIP_2_ is exposed for cleavage by PLC_303_ It is uncertain whether S phosphorylation by PKC comes before increased Ca^2+^ concentration from Trop2 signaling or after or whether this phosphorylation itself releases PIP_2_ [4]. When PLC cleaves PIP_2_, this results in an increase of IP_3_ (inositol 1,4,5-triphosphate) in the cytoplasm and DAG (deacylglycerol) in the plasma membrane. IP_3_ causes Ca^2+^ release from the endoplasmic reticulum (ER) [82]. The increase in free Ca^2+^ and DAG could activate more PKC in a positive feedback mechanism. This increase in PKC could lead to further phosphorylation of Trop2 and activation of the Raf and NF-κB pathways [4]. Ca2+release stimulates MAPK signaling and cell cycle progression [8].

The incomplete or aberrant production of Trop2 may cause it to lose its function and to be internalized from the membrane into the cytoplasm, where it plays a role in cancer progression [7]. In GDLD, an accumulation of Trop2 is observed in the Golgi apparatus due to a defective transport [3]. When PIP_2_ is bound to the cytoplasmic tail of Trop2, phospholipase C (PLC) cleavage is important for Ca^2+^ release and for Trop2-mediated cell signaling and cell cycle progression [8]. Figure 2 shows this pathway. The exact mechanisms of Trop2 regulation and cell signaling have not been elucidated.

Trop2 is reputed as a prognostic factor and a marker for numerous cancers. Trop2 mutations have been directly linked to GDLD but there are no known Trop2 mutations that have been implicated in cancers [3]. Trop2 has been reported to be overexpressed in the following solid tumor cancers: breast, cervix, colorectal, esophagus, gastric, certain lung cancers, squamous cell carcinoma of the oral cavity, ovary, pancreas, prostate, stomach, thyroid, urinary bladder, and uterus [11]. However, it has been reported to be underexpressed in non-small lung cancer [7]. Trop2 is upregulated in several hematologic malignancies such as leukemia, extranodal nasal type lymphoma (ENK/TL), and Non-Hodgkin's lymphoma (NHL), whereas no Trop2 expression is found in anaplastic large cell lymphoma (ALCL) [12,13]. Currently, several antibodies, antibody drug conjugates, and inhibitors have been reported to target Trop2 expression with the objective of decreasing Trop2 overexpression and, thus, decreasing tumor progression in certain cancers (see below).

### The *Trop2* Gene

The gene that codes for Trop2 is called *Tacstd2,* Tumor-associated calcium signal transducer 2, and it is found on chromosome 1p32 [2]. It is about 35 kDa, with some sources documenting the size as 35.7 kDa and 35.79 kDa [3]. *Tacstd2* may be genetically and epigenetically regulated (see below for details). It has a CpG island, which is a DNA sequence of at least 200 base pairs with GC content above 50% and a ratio of CpG dinucleotide above 0.6, which covers a DNA fragment larger than 4 kb and is located upstream of the transcriptional start site. *Tacstd2* has 78 CpGs and 4 SP1 sites (GGGCGG) [7].

Trop2 is composed of 323 amino acids [7]. MARGPGLAPPPLRLPLLLLVLAAVTGHTAAQDNCTCPTNKMTVCSPDGPGGRCQCRALGSGMAVDCSTLTSKCLLLKARMSAPKNARTLVRPSEHALVDNDGLYDPDCDPEGRFKARQCNQTSVCWCVNSVGVRRTDKGDLSLRCDELVRTHHILIDLRHRPTAGAFNHSDLDAELRRLFRERYRLHPKFVAAVHYEQPTIQIELRQNTSQKAAGDVDIGDAAYYFERDIKGESLFQGRGGLDLRVRGEPLQVERTLIYYLDEIPPKFSMKRLTAGLIAVIVVVVVALVAGMAVLVITNRRKSGKYKKVEIKELGEL RKEPSL

Trop2 is part of the GA733 family, which is composed of GA733-1 (Trop2) and GA733-2, which is also known as EpCAM (epithelial cell adhesion molecule). Trop2 might be a modulator and/or an enhancer of EpCAM signaling [4]. Trop2 is intronless, while EpCAM is not and has 9 exons [14]. Trop2 and EpCAM have a very high structural similarity and a high sequence similarity; Trop2 has about 49% homology with EpCAM [9]. They are both type I transmembrane proteins with single transmembrane domains [14]. EpCAM has a role in cell-cell adhesion and cell signaling through c-myc and cyclin ATE, proliferation, migration, invasion, and differentiation. EpCAM overexpression has been correlated with poor prognosis in breast and ovarian cancers, but with increased survival in renal cell carcinoma [4]. In small-sized adenocarcinomas, Trop2 and EpCAM have opposite biological effects: Trop2 has an unfavorable outcome, while EpCAM has a favorable one [14].

### Trop2 Domains

Trop2 is composed of several domains that span the cell membrane. It starts with a hydrophobic leader peptide that is made up of the first 26 amino acids [4]. The largest part of the molecule is the N-terminal extracellular part, also known as the ectodomain (Trop2EC). It is composed of 3 domains, anchored via a single transmembrane helix (TM) followed by a short intracellular tail of 26 amino acid residues (Trop2IC). The Trop2EC is composed of a small N-terminal cysteine-rich domain (CRD) as well as a cysteine-poor domain (CPD) [9]. Trop2 is predicted to have 4 N-linked glycosylation sites at the residues 33, 120, 168, and 208 [4]. An extracelullar EGF (epidermal growth factor)-like repeat domain is made up of the amino acids 1-274 [4,7]. A thyroglobulin type-1 domain (TY) is located between amino acids 70-145, within the extracellular domain [4].

Trop2 extracellular domains (EC) contain globular portions with the GA733 type-I domain and a thyroglobulin type-IA motif, which are required for Trop2 homomultimerisation. The cysteine free region acts as a stem to connect a globular moiety to single hydrophobic transmembrane segments. The short (25-26 amino acid long) intracytoplasmic C-terminal tail appears devoid of enzymatic domains.

The amino acids 275-297 make up the transmembrane domain (TM). Lastly, Trop2 has a cytoplasmic tail that has a phosphatidylinositol 4,5-bisphosphate (PIP_2_)-binding motif, a conserved tyrosine site, and a serine phosphorylation site at S_303_. Part of Trop2 signaling is dependent on protein kinase C (PKC) and involves phosophyorylation of S_303_ [4].

The Trop2EC and Trop2IC are cleaved off of the TM in the process of regulated intramembrane proteolytic cleavage. The released Trop2IC indirectly influences cell proliferation and self-renewal through the β-catenin signaling pathway (see below) [9].

### Trop2 Expression and Function in Normal Tissues

#### A. Role in Embryogenesis

Trop2 is expressed in trophoblast cells, which possess the capacity to invade uterine decidua during the process of placental implantation. The protein is highly expressed in these cells, which explains the origin of the Trop2's name [4]. Trop2/*Tacstd2* is a marker highly enriched in spheroids and is expressed throughout the embryonic-day-14 intestinal epithelium. It is highly expressed in all epithelial proliferating progenitors of the E14 duodenum. *Tacstd2* is among the most upregulated genes in spheroids versus organoids. At E15.5, Trop2 is expressed in cells at the tip of the newly formed villi and the ileum, with a lower yet still detectable signal in the intervillus zone. Between E16.5 and birth, Trop2 expressing cells progressively disappear, most likely by being shed from the villi [15].

In one report in which mTrop2/EpCAM is knocked out in mice, the mice were born alive, but they died soon after birth because of an inability to gain weight as well as experiencing hemorrhagic diarrhea, thus showing the necessity of Trop2 in early development and survival [16].

#### B. Role in Fetal Growth

Trop2 has been found to be upregulated during accelerated fetal lung growth/expansion. In one study, an induced 75% reduction in Trop2 expression led to a 50% decrease in the percentage of proliferating fibroblasts. Thus, Trop2 may have a role in regulating normal fetal lung growth. As organogenesis and tumorigenesis are thought to share common regulatory pathways, it is hypothesized that Trop2 may promote cell proliferation in the developing lung.[1] Trop2/Cnx43+ cells act as transient stem cells responsible for the generation of fetal intestine in an environment with low Wnt and high Bmp stimulatory tones, which prevail at this period of intestinal development [15].

#### C. Expression in Normal Tissues

Trop2 is expressed in a number of normal tissues, which is important to note when considering the targeting of Trop2 expressing cancer tissues. In fetal rat lungs, Trop2 is mainly expressed in type II alveolar epithelial cells (AECs), interstitial fibroblasts, smooth muscle cells, myofibroblasts, and airway epithelial cells [1]. Membrane localized expression of Trop2 has been noted in stratified squamous, cuboidal, and columnar epithelial cells. Trop2 has been expressed, albeit at different levels, in the following normal (non-cancerous) tissues: the epithelial barrier/lining of the stratum basale epidermis, breast, cervix, cornea, the epithelial secretory tissue of the endocrine and exocrine glands, esophagus, heart, kidneys (distal convoluted tubules and collecting ducts), larynx, lung, liver, pancreas, prostate, salivary gland, skin, thymus, tonsils, trachea, trophoblast cells, urothelium, and uterus [11].

### Trop2 Binding Partners

Several reports have shown that Trop2 binds to several factors, such as IGF-1, claudin-1 and 7, cyclin D1, and PKC, and these are briefly described below.

#### A. IGF-1

IFG-1 (insulin-like growth factor 1) might be a ligand of Trop2, allowing it to activate its putative downstream mediators (PIP_2_ and Ca^2+^ ) and modulate IGF-1R signaling. Trop2's phosphatidylinositol 4,5-bisphosphate (PIP_2_)-binding domain contains a phosphorylation site (S_303_) and an extracellular EGF-like and thyroglobulin type-1 repeat domain. Trop2's PIP_2_binding domain may bind IGF-1 and, thus, outcompete the IGF-1 binding protein. Another possibility for Trop2's function is that it may form a complex with IGF-1 and, thus, prevents IGF-1R signaling [7].

#### B. Claudin-1 and Claudin-7

The transmembrane proteins claudin-1 and claudin-7 are binding partners of the Trop2 ectodomain (EC). They play important roles in tight junctions at the epithelial barrier. Trop2 is speculated to act as an anchor or as a transporter during claudin rearrangement or it may function as a stabilizer to prevent claudin degradation via the ubiquitin-proteasome system [9].

Trop2 is important in maintaining the tight junction integrity as well. Trop2 might indirectly affect adhesive interactions between proteins because it can modulate cell adhesion to fibronectin through the P1 integrin/RACK1 (receptor for activated protein kinase C) complex formation [9]. Loss of Trop2 leads to decreased expression and rearrangement of the subcellular localization of certain proteins, which affect the performance of the epithelial barrier. This is specifically noted in the GDLD. The claudin-4 protein is thought to reside closely next to Trop2, via a side-by-side or head-to-head interaction with the claudin-1 or claudin-7 protein, but it does not bind Trop2. Trop2 and claudins-1 and -7 are thought to bind to each other through their AxxxG motifs because EpCAM binds claudin-7 through this interaction. The exact mechanism is unclear [17].

#### C. Cyclin D1

Trop2 forms an oncogenic fusion protein with cyclin D1 [18]. Bicistronic cyclin D1-Trop2 mRNA arises from the posttranscriptional joining of the cyclin D1 transcript at the poly-adenylation site in exon V with the full-length Trop2 mRNA, leading to the independent expression of both proteins. The appearance of the bicistronic mRNA occurs by the intermolecular splicing in trans of the two mRNAs. The binding of the 2 mRNA molecules increases the stability of cyclin D1 when compared to its normal-full length transcript. This chimera is expressed by human tumors differentially. Low amounts of the chimera are enough to increase the life span and cell proliferation of senescent primary cells [4]. The chimeric cyclin D1-Trop2 protein is implicated in cell transformation; silencing this fusion protein inhibits tumor growth [7].

#### D. PKC

The HIKE region of Trop2 binds to protein kinase C (PKC) and is phosphorylated by PKC. HIKE is a highly conserved sequence motif identified as a candidate pleckstrin-homology (PH) domain binding site in GP proteins, protein kinases, ankyrin and kinesin. PKC regulates the binding of downstream effectors to target sequences of phosphorylation at S/T residues [3]. PKCa is an isoform of PKC that interacts with the tail of Trop2. It is activated by Ca^2+^ and deacylglycerol (DAG) and is translocated to the plasma membrane. Activation of PKCa is necessary for podosome formation.

PKC phosphorylates Trop2's cytoplasmic tail at the S_303_. PIP_2_(phosphatidylinositol 4,5-bisphosphate) has been suggested to regulate the phosphorylation of S_303_ by PKC. If the cytoplasmic tail of Trop2 is bound to PIP_2_, it might be concentrating Trop2 for hydrolysis by phospholipase C (PLC). When position S_303_is activated or phosphorylated, the binding of the cytoplasmic tail to PIP_2_ might be reversed. This exposes PIP_2_ for cleavage by PLC, which results in an increase of IP_3_ (inositol 1,4,5-trisphosphate), DAG (deacylglycerol), and Ca^2+^ release from the endoplasmic reticulum. The increase in free Ca^2+^ and deacylglycerol could activate more PKC in a positive feedback mechanism, which could lead to the phosphorylation of more Trop2 and the activation of the Raf and NF-κB pathways by PKC (Figure 2). It is uncertain whether the phosphorylation of S_303_ comes_Ca_2+ before or after the increased concentration from Trop2 signaling or whether the phosphorylation of S_303_ releases PIP_2_ [4].

### Cell Signaling Mediated by Trop2

Trop2 upregulation drives the expression and activation of CREB1 (cyclic AMP-responsive element binding protein), Jun, NF-κB, Rb, STAT1 and STAT3 through induction of the cyclin D1 and the ERK (extracellular signal regulated kinase)/MEK (MAPK/ERK kinase) pathways. Growth-stimulatory signaling through NF-κB, cyclin D1 and ERK were shown to require an intact Trop2 cytoplasmic tail; its deletion abolishes stimulation of growth and modulation of NF-κB, cyclin D1 and pERK [19].

Trop2 has a HIKE-like phosphoinositide-binding motif, which is frequently present in signal transducers and can act as a docking site for regulatory/effector molecules [19]. The HIKE region (highly conserved sequence motif identified as a candidate pleckstrin-homology (PH) domain binding site in GP proteins, protein kinases, ankyrin and kinesin) may have a regulatory role in protein-protein and protein-lipid bindings [3].

Trop2 causes activation of the ERK1/3-MAPK pathways, which both govern cell cycle progression and may protect cancer cells from apoptosis [7]. Trop2 might indirectly affect adhesive interactions since it can modulate cell adhesion to fibronectin through the P1 integrin/RACK1 complex formation [9].

Trop2 potentially plays a role in deregulating characteristic stem cell proliferation and differentiation pathways such as Notch, hedgehog, and Wnt [8]. Trop2 functionally regulates adult tissue self-renewal and prostate regeneration [10]. Aberrant glycosylation of CD133 or Trop2 in prostate cancer stem cells may affect their folding and stability, resulting in incorrect protein assembly and abnormal signaling networks, thus promoting cancer growth [20]. mTrop2 expression leads to increased levels of phosphorylated p42/p44 MAPK (ERK1/ERK2), which are master regulators of the transition from the G1 to the S-phase. Cyclin D1 and cyclin E are downstream targets of the ERK/MAPK pathway and they are involved in the termination of the G0-G1 cell cycle arrest and the initiation and progression of the S phase. Thus, mTrop2 expression results in increased cell proliferation at low serum concentrations with an increased percentage entering the DNA synthesis phase [8].

Podosomes are actin-rich adhesion structures that control the activity of matrix metalloproteinases. They are thought to contribute to matrix remodeling and to serve as adhesion locations for invasive cells. Activation of Trop2 could indirectly lead to podosome formation by increasing the intracellular calcium concentration required to activate PKCa which could then phosphorylate Trop2 and signal via Src, Cdc42, and RhoaA to induce the formation of podosomes [4].

#### A. Trop2-Mediated MAPK Signaling

**Figure 3:**
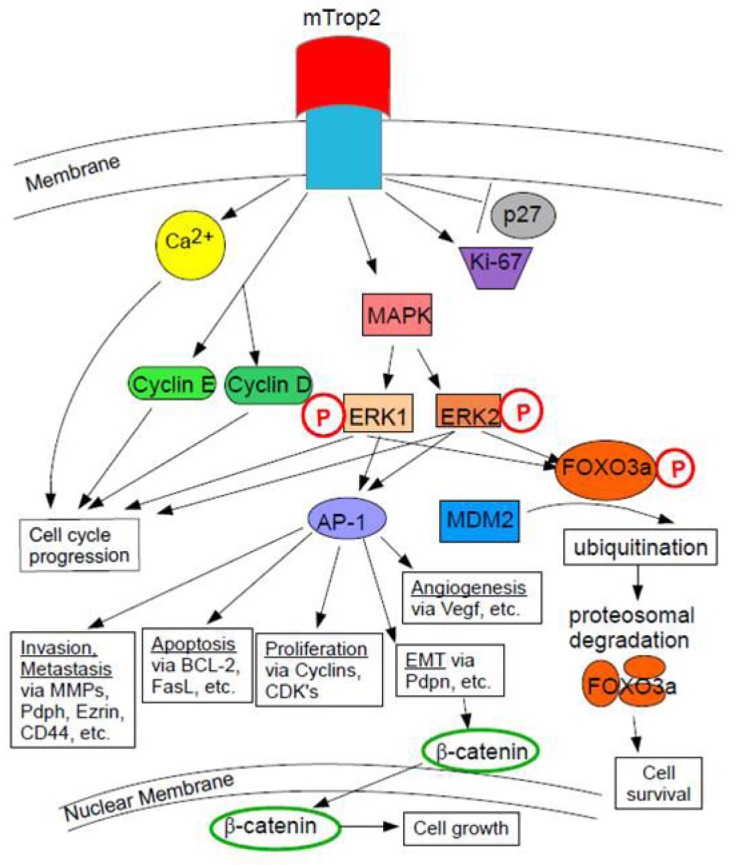
mTrop2 Cell Signaling and Resulting Activities mTrop2 expression increases the expression of the proliferation marker Ki-67 and causes Ca^2+^ to be mobilized from internal stores. mTrop2 expression downregulates p27 (cyclin-dependent kinase inhibitor 1B). mTrop2 expression activates MAPK signaling, which increases levels of phosphorylated ERK1 and ERK2. MAPK signaling and cell cycle progression can be further stimulated by Ca^2+^. mTrop2 increases levels of cyclin D1 and cyclin E, which help mediate ERK1/2 cell cycle progression (an increased percentage of cells enter the S phase). ERK signaling leads to induction of the AP-1 transcription factor [8]. It is a central regulator of tumor-associated target genes during carcinogenesis. AP-1 causes angiogenesis via VEGF (vascular endothelial growth factor), cell proliferation via the cyclins and CDKs, apoptosis via pro-apoptotic bcl-2 (B-cell lymphoma 2) or FasL (Fas ligand), and causes cell invasion and metastasis via MMPs (matrix metalloproteinases), Pdpn (podoplanin), Ezrin, and CD44, and it causes the epithelial to mesenchymal transition (EMT) via Pdpn. The EMT allows for the nuclear translocation of β-catenin, which causes cell growth via β-catenin's downstream effectors [26]. Heightened ERK activity could induce phosphorylation of FOXO3a at residues S294, S344, and S245, which can lead to ubiquitination by MDM2 (mouse double minute 2) and subsequent cytoplasmic localization and proteasomal degradation [8]. FOXO3a can induce cell death, therefore, its degradation could help promote cell survival in cancer [39].

mTrop2 expression can lead to MAPK signaling activation. The MAPK pathway can be further stimulated by an increase in Ca^2+^. MAPK signaling results in the induction of the AP-1 transcription factor and the downregulation of p27, which is a cyclin-dependent kinase inhibitor 1B. P27 binds to and prevents activation of cyclin D1-CDK4 to cyclin E-CDK4 complexes. Expression of mTrop2 is correlated with increased expression of the proliferation marker Ki-67. Downstream targets of MAPK are cyclin D1 and cyclin E. Figure 3 demonstrates the Trop2-mediated MAPK signaling pathway. mTrop2 increases the levels of phosphorylated MAPK (ERK1/ERK2) and, thus, mediates cell cycle progression. Increased levels of cyclin D1 and cyclin E help mediate cell cycle progression. An increase in intracellular calcium from internal stores could have an effect on cell signaling activation and on the activation and progression of the cell cycle through activation of PKC and/or the calcium/calmodulin-dependent protein kinase II (CaMKII). An increased percentage of cells, thus, enters the S phase. Activation of ERK is observed in several cancers and in cells that overexpress human Trop2. Heightened ERK activity could induce the phosphorylation of FOXO3a at residues S294, S344, and S425. This can cause its ubiquitination by MDM2, which then promotes FOXO3a's cytoplasmic localization and proteasomal degradation. The interaction between the ERK pathway and FOXO3a is reported to promote cell growth and tumorigenesis. It is uncertain whether Trop2-induced activation of ERK directly results in FOXO3a degradation. ERK1/2 activation could be providing anti-apoptotic signals which promote tumor cell survival [8].

#### B. Trop2-Mediated IGF-1R Signaling in Lung Cancer

High Trop2 expression attenuates IGF-1R signaling, which suppresses lung cancer growth and malignancy. IGF-1 might be a ligand of Trop2, allowing it to activate downstream mediators (PIP_2_ and Ca^2+^) and modulate IGF1R signaling. Another possibility is that the extracellular EGF-like and thryoglobulin type-1 repeat domains of Trop2 may form a complex with IGF-1 and, thus, mediates/suppresses IGF-1R signaling. During lung development, Trop2 acts as an attenuator of the IGF-1/IGF-1R signaling pathway whereby the ECD competes with the IGF-1 for IGF-1 binding [9]. Trop2 downregulates signaling by trapping IGF-1 in the surrounding microenvironment and, thus, outcompeting IGF-1R, which inhibits activation of IGF-1R signaling-mediated gene expression.

Trop2 signals through β-catenin (downstream targets cyclin D1 and c-myc get upregulated). Trop2's function requires the presence of β-catenin. β-catenin colocalizes with ICD within the nucleus. This is restricted to cancer regions and does not occur in benign tissues [10].

Downstream of this IGF-1/IGF-1R downregulation, the *AKT/ERK* gene, β-catenin, and slug expressions are suppressed. Trop2 may regulate activites of AKT and ERK depending on the histotypes of cancer cells. When there is less Trop2 expression on the membrane, this could reduce IGF-1R signaling-mediated AKT/β-catenin /slug expression and ERK activation through a direct binding of IGF-1 [7].

### Transcriptional Regulation of Trop2 Expression

#### A. Transcription factors of Trop2

##### 1. CREB1

The *Tacstd2* gene has a switch-like behavior, with CREB1 as a possible regulator. CREB1 is activated in breast cancer and modulates the transcription of *Tacstd2.* At the same time, the activation of *Tacstd2* increases the cytoplasmic calcium (Ca^2+^) level, which could in turn activate CREB1 through calmodulin-dependent protein kinases (e.g. CaMKII). The activated CREB1 could bind to the promoter of *Tacstd2,* and form a positive feedback system to promote and maintain the on state of *Tacstd2* [21].

##### 2. NF-κB

NF-κB and Trop2 have regulatory roles on each others' expression levels. Experiments in human breast cancer cell lines have shown that Trop2 gene expression is downregulated within a couple of hours of either the inhibition of NF-κB's protein activity or by its reduction in translocation, which confirms a regulatory role of NFκB on the transcription of the *Trop2* gene. There is, thus, a potential positive feedback system between NF-κB and Trop2 that supports the ‘switching’ behavior of Trop2 expression, which was uncovered in a previous analysis [21].

##### 3. HOXA_10_

HOXA_10_ (homeobox) is known to downregulate Trop2 expression [22]. It is involved in the proliferation of hematopoietic stem cells and progenitor cells. Its over-expression is associated with cancer development and poor prognosis in patients with acute myeloid leukemia and solid cancers [23].

The HOXA_10_ promoter has an NF-κB-binding site. NF-κB expression can lead to inflammation and carcinogenesis [23]. Trop2 phosphorylation can lead to downstream NF-κB activation [4]. Thus, it is possible that Trop2 downregulation by HOXA_10_ may decrease NF-κB activation, which can thus decrease the NF-κB-binding to the HOXA_10_ promoter, which could lead to a decrease in cancer progression.

#### B. Trop2 expression is further modulated by the inactivation of the following transcription factors

##### 1. TCF-1/LEF-1/HNF1A

The Lymphoid enhancer factor, (LEF1)/T-cell factor (TCF-1)/hepatocyte nuclear factor 1 (HNF1A) acts as a feedback transcriptional repressor of the *β-catenin*-TCF-4 target genes. Disruption of this negative feedback induces the formation of intestinal tumors. HNF1A/TCF-1 is the largest hub in the Trop2 transcription network and is essential for stem-cell growth [19].

When appropriate extracellular signals are delivered to an epithelial cell, β-catenin accumulates in a form that is able to be transported to the nucleus, where it can associate with hTCF-4 (a form of TCF expressed in normal colonic epithelium). Interaction of hTCF-4 with β-catenin results in transcriptional activation. The genes that are activated—possibly *Trop2*—may participate in the generation and turnover of epithelial cells. The disruption of β-catenin-TCF signaling may be an early step in malignant transformation [24]. If the accumulated β-catenin is found in the nucleus, it may potentially bind Trop2 and lead to cancer progression, suggesting that TCF disruption may be a positive regulator of Trop2.

##### 2. TP63/TP53L

The tumor protein 63 (TP63)/tumor protein 53L (TP53L), a homologue of the tumor suppressor p53 drives the expression of genes involved in cell adhesion, proliferation, and death [19]. p63 is a positive prognostic factor in cancer progression and outcome. The predominant isoform, ßNp63α, can act as a transcriptional repressor, possibly of Trop2. It is overexpressed in a number of epithelial cancers. By maintaining the epithelial character of cancer cells, ßNp63α may act like a supressor of cancer metastasis, which is a similar role of normal Trop2 expression [8,25]. At the same time, ßNp63α may possibly act to promote early steps in tumorigenesis by protecting cells from growth arrest and apoptosis, yet still allowing cells the plasticity necessary for physiologic and pathologic epithelial to mesenchymal transitions (EMT) [25]. The EMT leads to loss of cell-cell adhesion, loss of apical-basal cell polarity, increased motility of cells, loss of epithelial markers such as E-cadherin, gain of mesenchymal markers such as vimentin, and acceleration of TGF-β signaling [26]. Activation of β-catenin signaling is another function. Loss of p63 expression predisposes to a loss of epithelial characteristics and acquisition of mesenchymal characteristics, resulting in up-regulation of genes associated with tumor invasion and metastasis [2].

##### 3. ERG

*ERG (ETS-related gene),* of the ETS (erythroblast transformation-specific) family, regulates endothelial cell-adhesion molecules and interleukin 8 [19]. It is required for embryonic stem cell (ESC) differentiation toward the endothelial fate and it regulates angiogenesis and endothelial apoptosis. The growth promoting effects of ERG suggest that aberrant regulation of ERG could play an important role in the development of leukemia and other cancers. ERG has 36,000 target regions and participates in a complex harboring several other transcription factors associated with normal hematopoietic development. Trop2 could possibly be a target gene of ERG. Post-translational modifications of ERG could lead to cancer progression [27]. Inactivation of ERG may have a correlation with upregulation of Trop2 in certain cases and, thus, cancer progression.

##### 4. GRHL3

GRHL-1/Get-1 (grainyhead-like epithelial transactivator) belongs to a large family of genes encoding developmental transcription factors. GRHL3 is expressed in human endothelial cells and is required for endothelial and epidermal cell migration, which is similar to the function of Trop2 [28]. When mutated, it affects multiple genes linked to terminal differentiation and possibly Trop2 [19]. GRHL3 activates ERK1 and ERK2—as does Trop2—which are known inductors of proliferation, in keratinocytes. GRHL3 overexpression increases Akt phosphorylation [28]. In lung cancer, Trop2 actually attenuates IGF-1R signaling-mediated AKT expression and, thus, has the opposite effect [7]. GRHL3 increases Akt and eNOS (endothelial nitric oxide synthase) activation, which are both required for apoptosis protection. Loss of GRHL3 may result in endothelial dysfunction, due to loss of its anti-apoptotic effect [28].

##### 5. PU.1

SPI1/PU.1 (spleen focus forming virus [SFFV] proviral integration oncogene) functions in germinal progenitors [19]. PU.1 is the hallmark ETS factor involved in hematopoiesis and is a regulator of gene expression during myeloid cell development [27]. It can modulate the expression of at least 3000 genes expressed in hematopoietic cells; there are more than 1000 direct target genes [29]. *Tacstd2* could possibly be one of them. AP-1 is a downstream target of Trop2 expression [8]. C-Jun, which is part of the AP-1 transcription factor complex, functions as a critical co-activator of PU.1 transactivation of various myeloid promoters. Dysregulation of PU.1 activity can contribute to leukemogenesis [29].

##### 6. WTl

WT1 (Wilms' tumor suppressor gene) is critical for urogenital development and has a complex role in tumorigenesis, partly through the regulation of the G2/M transition [19]. WT1 suppresses cyclin E, whereas mTrop2 increases levels of cyclin E [8,30]. Trop2 is a calcium signal transducer and WT1-associated protein is a regulator of Ca^2+^ homeostasis [19,31]. WT1 directly binds to the AKT promoter and creates a positive feedback loop between WT1 and AKT expression via the P13K/AKT pathway in lung cancer. High levels of WT1 IgG antibody expression in lung cancer is associated with a worse prognosis, but when Trop2 is increased in lung adenocarcinomas, it can suppress cancer progression, suggesting downregulation [7, 23]. In Wilms' tumor, WT1 suppresses the IGF-1R gene, while Trop2 lowers IGF-1R levels in lung cancer [7,33].

##### 7. Glis2

Glis2 is a Kruppel-like zinc finger transcription factor involved in kidney development; loss of it causes nephronophthisis through the induction of increased apoptosis and fibrosis [19]. Glis proteins have been hypothesized to be activated through post-translational modifications and subsequently translocated to the nucleus where they regulate transcription by interacting with Glis binding sites in the promoter regions of target genes [34].

Glis2 may act as a repressor of epithelialmesenchymal transition (EMT). Glis2 acts as a negative regulator of β-catenin and inhibits TCF/LEF signaling and the β-catenin-TCF/LEF mediated activation of cyclin D1 [34]. The nuclear ICD of Trop2 colocalizes with β-catenin in the nucleus and is involved in a cascade that upregulates cyclin D1 [10]. Thus, Trop2 and Glis2 have opposite effects on β-catenin. Lack of Glis2 may enhance the transcriptional activation of β-catenin and, therefore, promotes tumorigenesis and allows for Trop2-β-catenin localization in the nucleus [35].

##### 8. AIRE

AIRE is an autoimmunity regulator transcription factor [19]. AIRE promotes T-cell tolerance and prevents autoimmune diseases. Deficiencies in AIRE cause elevated T-cell immune responses, which serve to suppress melanoma outgrowth [36]. If AIRE deficiencies allow cells to be more aggressive in their attacks and less tolerant, this may have a negative effect in some cancers. AIRE complexes with and influences the activity of many transcription factors. It interacts with the common coactivator CREB-binding protein (CBP), which is known to upregulate Trop2 [37,38]. By activating CBP, AIRE may be, thus, regulating Trop2, although it is not certain.

##### 9. FOXM1

FOXM1 (forkhead box protein transcription factor) regulates G2-M transcription [19]. *FOXM1* has been identified as one of the most commonly upregulated genes in human solid tumors [39]. It has stem cell-like properties, as does Trop2. Phosphorylation of FOXM1 via the RAF/MEK/MAPK pathway stimulates FOXM1. Nuclear FOXM1 is functionally activated in tumor cells. Trop2's downstream ERK and Raf pathways could phosphorylate FOXM1, suggesting a positive feedback relationship [8].

FOXM1 knock-down leads to decreased expression of p27, while Trop2 expression downregulates p27 [8,39]. FOXM1 overexpression contributes directly to metastatic behavior by driving the EMT through AKT [39]. In lung tumorigenesis, Trop2 actually attenuates AKT expression and its downstream targets [7]. FOXO3a negatively regulates FOXM1 target gene expression. Nuclear accumulation of FOXO3a can repress FOXM1 and induce cell death [39]. Trop2 downstream effectors can induce phosphorylation of FOXO3a. Heightened ERK activity could lead to a pathway that ends in proteasomal degradation of FOXO3a, which would then prevent the negative regulation of FOXM1 by FOXO3a [8].

##### 10. FOXP3

FOXP3 is another forkhead box protein transcription factor that is associated with metastatic disease. Inactivation of FOXP3 modulates Trop2 expression via an indirect mechanism [19].

### Expression of Trop2 in Cancer and its Prognostic Significance

Overexpression of wild-type Trop2 is shown to be necessary and sufficient to drive cancer growth [40]. Trop2 is implicated in the activation of the ERK/MAPK pathway, leading to downstream alterations in cellular proliferation, migration, invasion, and survival of cancer cells [41]. Trop2-driven signaling is essential for the growth of human cancer cells *in vitro* and in pre-clinical models [19]. It has a role in accelerating the cancer cell cycle and cell growth stimulation [14].

Trop2 overexpression is associated with decreased patient survival as well as increased tumor aggressiveness and metastasis in many cancers. EpCAM and Trop2 are similarly expressed in many small-sized pulmonary adenocarcinomas: the expression of EpCAM is significantly related to a favorable outcome, while Trop2 expression tends to indicate an unfavorable outcome [14].

Trop2 is overexpressed by various human carcinomas including, breast, cervix, colorectal, esophagus (some types), lung (some types), nonHodgkin's lymphoma, chronic lymphocytic lymphoma (CLL), Raji Burkitt lymphoma, oral squamous cell, ovarian, pancreatic, prostate, stomach, thyroid, urinary bladder, and uterine. Trop2 is not upregulated in the majority of esophageal, head, neck, and lung tumors and is not expressed in anaplastic large cell lymphoma (ALCL) [11,42]. Table 1 summarizes Trop2 expression in relation to specific cancers and its prognostic significance. Briefly, we present below the expression of Trop2 in various cancers:

**Table 1:** Trop2 Expression In Cancer

Cancer	Trop2 Expression	Prognostic Significance [References]
Anaplastic large cell lymphoma (ALCL)	No expression, implicating that its expression may not be involved in tumor growth	No [13]
Breast	Overexpression in some types ; downregulated in others	Yes [18, 7]
Cervical carcinoma	Overexpression	Suggested [44]
Colon cancer	Overexpression	Yes [46, 11, 7, 45]
Colorectal carcinoma	Overexpression	Yes [14, 47]
Endometrioid endometrial cancer (EEC)	Overexpression; higher tumor grade and cervical involvement	Yes [2]
Esophagus	Overexpression	Suggested [48, 11]
Gastric cancer	Overexpression	Yes [49]
Glioma	Overexpression	Yes [50]
Head and neck squamous cell carcinoma	Not upregulated on tumors	No [11]
Hilar cholangiocarcinoma	Overexpression	Yes [51]
Kidney	mRNA expression is downregulated	Suggested [7]
Large intestine	mRNA expression is upregulated	Suggested [7]
Lung and non-small cell lung cancer (NSCLC)	Downregulated in most lung cell lines	Yes, low Trop2 expression is significant [7, 11, 55]
Chronic lymphocytic lymphoma (CLL)	Overexpression	Possible [56]
Extranodal NK/T-cell lymphoma, nasal type (ENKTL)	Overexpression	Yes [57]
Non-Hodgkin's lymphoma (NHL)	Overexpression	Possible [12]
Small-sized Pulmonary adenocarcinoma	Overexpression	Yes [14]
Squamos cell carcinoma of the oral cavity	Overexpression	Yes [54, 14]
Ovarian	Overexpression	Yes [59, 11]
Pancreatic	Overexpression	Yes [60, 11]
Prostate	Overexpression	Yes [11, 7, 6, 62]
Stomach carcinoma	Overexpression	Suggested [11]
Thyroid carcinoma	Overexpression	Suggested [11]
Urinary bladder carcinoma	Overexpression	Suggested [11]
Uterine	Overexpression	Suggested [11]

#### A. Anaplastic large cell lymphoma (ALCL)

The absence of Trop2 in ALCL cells may indicate that it is not involved in tumor growth. Aberrant copies of the CCND1 / chromosome 11 may be observed in ALCL, probably as a consequence of the reported ploidy changes in these tumors. Aberrant cyclin D1 expression seems to promote proliferation in other types of lymphoma, while a growth promoting CCND1/Trop2 fusion product has also been described in these tumors. In ALCL, the ectopic expression of Trop2 would potentially indicate the presence of the CCND1/Trop2 fusion. However, since Trop2 is not present, therefore, ALCL tumors do not have CCND1/Trop2 fusion products [49]. The absence of Trop2 expression suggests that its expression may not be involved in tumor growth [13].

#### B. Breast cancer

Overexpression of Trop2 is found in BT272 breast cancer cells. ER-negative/HER2-positive tumors have higher Trop2 levels than ER-positive/HER2-negative cases. Lower levels of Trop2 are found in SKBR3, MDAMB-435, and MDA-MB-468 breast cancer cell lines [18]. The cyclin D1-Trop2 mRNA is a potent oncogene as it transforms primary cells *in vitro* and induces aggressive tumor growth *in vivo* in cooperation with activated RAS. Silencing of the chimeric mRNA inhibits the growth of breast cancer cells. The stabilized cyclin D1 mRNA cooperates with Trop2 in stimulating the growth of cancer cells [43]. Trop2 overexpression may be associated with a less favorable breast cancer phenotype [18].

#### C. Cervical cancer

Trop2 expression in cervical carcinoma is upregulated, but its expression is not vastly different from the Trop2 expression in normal cervical tissues [11]. The tumorigenic function of Trop2 in cervical cancer is associated with increased expressions of cyclin D1, cyclin E, CDK2, and CDK4, but reduced expressions of p27 and E-cadherin via the activation of the ERK signaling pathway. Overexpression of Trop2 is closely related with the FIGO stage, histological grades, lymphatic metastasis, invasive interstitial depth and high expression of Ki-67. Significantly decreased overall survival and progression free survival have been noted in patients. Trop2 may be a prognostic indicator for cervical cancer [44].

#### D. Colon and colorectal cancer

Trop2 is overexpressed in colon and colorectal cancer [11,14]. Trop2 expression leads to decreased survival in colon cancer.[7]. High expression can indicate poor prognosis [45]. Trop2 is a significant predictor of poorer patient survival and relates to the chance of disease recurrence and liver metastasis in colon cancer [46]. Expression in left-sided colon cancer (LSCC) is much higher than in right-sided colon cancer (RSCC) and may, thus, be a potential independent prognostic factor of LSCC [45]. Trop2 expression is highly expressed in colorectal tumors and is associated with an unfavorable outcome also [47].

#### E. Endometrioid endometrial cancer (EEC)

Trop2 may be clinically useful in the attempt to identify patients at higher risk of relapse before surgery, and subsequently, to optimize follow-up treatments. Trop2 is highly expressed in EEC. Its expression is much higher in poorly differentiated EEC as opposed to well-differentiated EEC. It is a marker for biologically aggressive tumor phenotypes and poorly differentiated EEC. It is an independent prognostic factor for poor disease-free survival and can prognosticate patient outcome [2].

#### F. Esophageal cancer

Trop2 expression is detected on esophageal carcinomas [11]. Protein levels are much higher in esophageal squamous cell carcinoma lines than in normal tissues and are notably higher in mild hyperplasia of esophageal mucosae. mRNA expression of Trop2 is not noted to be elevated in cancer tissues or cell lines [48].

#### G. Gastric cancer

Trop2 is overexpressed in gastric cancer. Trop2 is an independent prognostic marker for disease recurrence in the intestinal type gastric cancer. In intestinal-type gastric cancer, Trop2 expression is correlated with shorter disease-free survival. Overall, Trop2 overexpression is predictive of overall survival and poor disease-free survival in lymph node positive patients [49].

#### H. Gliomas

Trop2 expression is overexpressed in gliomas. Trop2 expression is much higher in WHO grade II and IV gliomas than grade II gliomas. Trop2 correlates with Ki-67 and microvessel density (MVD), but not with age or gender in gliomas. Trop2 expression is correlated with malignancy, proliferation, and angiogenesis in gliomas and tends to increase with increasing WHO grades [50].

#### I. Squamous cell carcinoma of the head and neck

Expression of Trop2 is not upregulated in head and neck tumors compared to normal tissues [11].

#### J. Hilar cholangiocarcinoma

Trop2 is expressed in hilar cholangiocarcinoma. Trop2 correlates with microvessel density as well. Trop2 expression has an independent prognostic value in patients with hilar cholangiocarcinoma. High Trop2 expression correlates with a much poorer overall survival rate than with low Trop2 expression [51].

#### K. Kidney cancer

Trop2 mRNA expression is downregulated in kidney cancer [7].

#### L. Squamous cell carcinoma of the oral cavity

Overexpression of Trop2 is found in oral squamous cell carcinomas [14]. The *Tacstd2* gene is a direct target of miR-125b-1, which causes dysfunction of the mitogenactivated protein kinase pathway (MAPK) by regulating *Tacstd2* expression. Loss of miR-125b-1 may have a key role in the pathogenesis and progression of squamous cell carcinomas of the head and neck and potentially other tumors [52]. Trop2 loss fails to suppress keratinocyte transformation, which causes keratinocytes to pass through an EMT and form tumors. Total loss of Trop2 protein expression is observed in the spindle cell component of sarcomatoid carcinomas [53]. Trop2 expression has an unfavorable outcome [14]. High expression correlates with aggressiveness and poor prognosis. It is an independent prognostic factor of poor disease outcome and it correlates with worse survival [54].

#### M. Large intestine

Trop2 mRNA expression is upregulated in cancer of the large intestine [7]. A cyclin D1-Trop2 mRNA chimera is expressed by many gastro-intestinal cancers [11].

#### N. Lung cancer

Trop2 expression is not upregulated on the majority of lung tumors [11]. Low Trop2 expression is observed in lung adenocarcinoma tissues as compared with their normal counterparts. This could be due to loss of heterozygosity (LOH) or hypermethylation of the CpG island DNA of Trop2 upstream of the promoter region. Inactivation of Trop2 may play a role in lung cancer tumorigenicity through losing its suppressive effect on IGF-1R signaling and tumor growth. *Tacstd2* is a potential tumor suppressor gene in lung cancer development. It has a potential suppressive role in malignancy [7].

Low expression of Trop2 is noted in in NSCLC in patients. It is downregulated in most lung cell lines except for H322M (adenocarcinoma), EKVX (adenocarcinoma), and HOP92 (large cell). Trop2 promotes cell proliferation and increases colony formation, thus resulting in cells increasing in volume. Downregulation of Trop2 by DNA hypermethylation or loss of heterozygosity (LOH) may lead to lung carcinogenesis [7]. Trop2 overexpression shows better survival in NSCLC in patients with adenocarcinoma and may be a better prognostic marker in advanced stage adenocarcinoma [55].

#### O. Lymphomas

Trop2 is overexpressed in chronic lymphocytic lymphoma (CLL), extranodal NK/T-cell lymphoma, nasal type (ENKTL), and non-Hodgkin's lymphoma (NHL) [12,56,57]. Trop2 is overexpressed in extranodal NK/T cell lymphoma, nasal type (ENKTL) and is associated with lymph node involvement and poor overall survival. Trop2 expression reflects a more malignant phenotype and may be an unfavorable prognostic factor for ENKTL [57].

#### P. Small-sized pulmonary adenocarcinoma

Trop2 overexpression is found in nonlepidic-type tumors. Besides the cell membrane, Trop2 is additionally expressed in the cytoplasm. Trop2 is associated with an unfavorable outcome and is an independent prognostic factor. It has the opposite effect of its homologue, EpCAM, which is associated with a favorable outcome. Trop2 expression is detected in more poorly differentiated tumors, which suggests that it may act as an oncogene [14].

#### Q. Ovarian cancer

Trop2 protein is overexpressed and Trop2 mRNA expression is upregulated in epithelial ovarian cancer [11]. Trop2 overexpression has been reported in serous ovarian cancer, a notably aggressive, treatment-resistant gynecologic malignancy. Trop2 expression was found in 82% of chemotherapy-resistant ovarian tumor tissues at mRNA and protein levels in one study. Eighty three percent of the ovarian cancer cell lines tested by qRT-PCR and flow cytometry demonstrated high Trop2 expression. Aberrant Trop2 expression may account for the enhanced invasive behavior and increased biologic aggressiveness of chemotherapy-resistant ovarian carcinomas [58]. Trop2 overexpression may correlate with an aggressive malignant phenotype for epithelial ovarian cancer and may be a novel prognostic factor [59].

#### R. Pancreatic cancer

Trop2 is overexpressed in pancreatic cancer [11]. High Trop2 expression is correlated with the development and malignancy of pancreatic cancer. It is associated with poor prognosis and could be a novel prognostic biomarker [60].

#### S. Prostate cancer

Trop2 mRNA expression is downregulated in prostate cancer [7]. Trop2 overexpression enhances directional cancer cell migration and is involved in the metastatic competence of prostate cancer cells [40]. It causes basal prostate cells to efficiently form spheres and express stem cell characteristics [61]. Trop2 is involved in the metastatic competence of prostate cancer cells. Trop2 inhibits prostate cancer cell adhesion to fibronectin (FN) and, thus, promotes prostate cancer cell migration on fibronectin (FN). It enhances directional prostate cancer cell migration. Thus, Trop2 regulates prostate cancer cell adhesion to FN through activation of the P(1) integrin-RACK1-FAK-Src signaling axis. Trop2 expression correlates with prostate cancer cell aggressiveness [62]. Prostate basal cells expressing high levels of Trop2 are able to efficiently form spheres and express stem cell characteristics [61].

#### T. Stomach carcinoma

Trop2 expression is upregulated [11]. The prognostic significance has not been reported.

#### U. Thyroid carcinoma

Trop2 expression is upregulated [11]. The prognostic significance has not been reported.

#### V. Urinary bladder carcinoma

Trop2 expression is upregulated [11]. The prognostic significance has not been reported.

#### W. Uterine cancer

Trop2 expression is upregulated in cancer of the uterus [11]. Trop2 overexpression has been reported in uterine serous papillary carcinoma (USPC), an aggressive, treatment-resistant cancer [58].

### Trop2 and Drug Resistance

Several reports have suggested that Trop2 expression regulates tumor cell resistance to therapeutic drugs. Postulated mechanisms by which Trop2 regulates resistance are briefly discussed below.

#### A. FOXM1 dysregulation

FOXM1 dysregulation is a major cause of tumorigenesis and drug resistance. It may possibly promote drug resistance by enhancing DNA damage repair. It is involved in cancer progression and has been identified as one of the most commonly upregulated genes in human solid tumors [39]. It is a transcriptional regulator of Trop2, thus, its dysregulation may potentially have an effect on Trop2 expression.

The accumulation of reactive oxygen species (ROS) can activate FOXM1 in a negative feedback loop to activate genes whose products are involved in antagonizing the oxidative stress. Tumor cells may hijack the negative regulation of FOXM1 by ROS in order to evade cytotoxic effects of chemotherapeutic drugs and to promote survival of resistant clones [39].

#### B. HOXA_10_ expression

Expression of HOXA_10_ (homeobox-leucine zipper) protein—another transcription factor of Trop2— has been associated with resistance to chemotherapy [23]. High expression of HOXA_10_ causes resistance to combined chemoradiotherapy of concomitant and adjuvant temozolomide and radiotherapy in gliobastoma [63].

#### C. Tamoxifen resistance

The activation of *Tacstd2* increases the cytoplasmic_Ca_2+ level, which could, in turn, activate CREB and the MAPK/ERK pathways through calmodulin-dependent protein kinases (e.g. CaMKII). The activated MAPK pathway can increase the expression of cyclin D1 and cyclin E as well as reduce the level of the CDK (cyclin-dependent kinase) inhibitor, p27, to promote cell proliferation. The activated CREB can bind to the promoter of *Tacstd2* and forms a positive feedback to promote and maintain the “on” state of *Tacstd2.* Resistance to the estrogen antagonist drug tamoxifen is associated with the high expression of c-Fos, AP-1 (activator protein 1) and pCREB activation, which could mediate a constitutive “on” state of *Tacstd2,* and thus could correlate with increased Trop2 expression [21].

#### D. Trastuzumab resistance

Trastuzumab is an anti-HER2 breast cancer drug. Monoclonal antibody resistance is associated with the dysregulation of p27 and activation of cyclin D/E. Activation of *Tastd2* could modulate this dysregulation. Trastuzumab resistance is associated with increased cyclin E levels. Trop2 expression is known to inhibit p27, which can increase cyclin E levels, and may thus be a cause of this resistance [21].

#### E. Sensitivity to gefitinib in non-small cell lung cancer cell lines

A non-small cell lung cancer cell line found to be gefitinib-resistant had low levels of Trop2 expression. Alternately, flow cytometry analysis suggests that high levels are present. The reason for this discrepancy is unknown [58].

#### F. Resistance in ovarian and uterine cancers

Certain primary ovarian cancer cell lines were found to be highly resistant *in vitro* to multiple chemotherapeutic drugs including carboplatin, cisplatin, paclitaxel, doxorubicin, ifosfamide, gemcitabine and topotecan [58]. Uterine and ovarian carcinosarcomas, which are notoriously resistant to multiple clinically available chemotherapeutic agents, can be made highly sensitive to immune-mediated cytotoxicity when effector cells are engaged by the Trop2-specific antibody, hRS7 [64].

#### G. Head and neck cancer resistance to gefitinib and erlotinib

Common markers of resistance for epithelial growth factor receptor tyrosine kinase inhibitor drugs such as gefitinib and erlotinib for head and neck squamous cell carcinomas (HNSCC) and non-small cell lung carcinomas (NSCLC) include genes associated with the EMT, such as *Trop2.* Increased protein expression of vimentin combined with the loss of E-cadherin, claudin-4, and claudin-7 were associated with gefitinib resistance in both HNSCC and NSCLC cell lines. The loss of the Ca^2+^-independent cell-cell adhesion molecules EpCAM and Trop2 in resistant lines was confirmed. The loss of Trop2 expression was more predictive of gefitinib resistance in NSCLC than HNSCC. Thus, the EMT may play a role in establishing gefitinib resistance for both HNSCC and NSCLC [65].

### Pre-Clinical and Clinical Studies Targeting Trop2

#### A. Preclinical anti-Trop2 therapeutics

*Tacstd2* is an oncogene with the potential as a therapeutic target, specifically for antibody-based therapy. Trop2 has restricted expression in normal tissues, therefore, anti-Trop2 therapeutics would be predicted to have limited toxicity, although there is still a possible risk of potential toxicity to healthy tissues that normally express Trop2 [11]. Its overexpression in metastatic tissues makes it an attractive and potential therapeutic target for late stage diseases [4]. Trop2 is overexpressed in primary tumors and has been proposed to be a marker for undifferentiated epithelial cells [7,19]. Table 2 summarizes *in-vivo* and *in-vitro* therapeutics.

**Table 2:** Trop2 Preclinical Cancer Therapeutics

Inhibitor/Agent	Cancer	Testing	Response	References
		*In Vitro*		
Antigen-presenting cells (APC)	Lymphoma	Transformed murine fibroblast and eptihelial cell lines	Stimulate growth and cytotoxic effects of Trop2-specific antitumor cytotoxic lympocytes (CTL)	[66]
Rap (frog RNase) fusion proteins: 22-rap, 20-rap, C2-rap, 74-rap, 14-rap, and E1rap	Lymphoma	Cell lines	100% cell killing; potential in inhbiting CLL and Raji Burkitt lymphomas	[12]
(−)-Epigallocatechin-3-gallate (EGCG)	Colorectal cancer	Cell lines	Supresses Trop2 expression	[47]
hRS7	Ovarian carcinosarcoma (OMMT), uterine carcinosarcoma (UMMT), endometrial endometrioid carcinoma (EEC), cervical carcinoma refractory	Cell lines	High level of immune mediated cell death in OMMT and UMMT; induces antibody-dependent cellular toxicity (ADCC) against OMMT, EEC, cervical cancer refractory	[59, 68, 67]
Nano drug delivery of apoptosis activator 2	Gastric adenocarcinoma	Cell lines	Increase apoptosis of cancer cells	[71]
5-Aza-2′-deoxycytidine	Lung Cancer	Cell lines	Elevates Trop2 expression, which suppresses cell proliferation and colony formation	[7]
Human Fab antibody against Trop2	Breast Cancer	Cell lines	Induces apoptosis and inhibits proliferation of cancer cells	[70]
		***In Vivo***		
131I-IMP-R4-hRS7	Breast cancer	Nude mice xenografts	Decreased tumor volume; complete remission in 45% of cases according to one study	[72]
2L-Rap(Q)-hRS7	Cervical, breast, colon, pancreatic, ovarian, non-small cell lung and prostate; NSCLC	Nude mice xenografts	Suppresses tumor growth	[73]
90Y-hPAM4 RAIT (radioimmunotherapy) with hRS7-SN-38 conjugate (ADC)	Non-Hodgkin's Lymphoma; pancreatic cancer	Nude mice tumors	Higher survival rate wit(100%) h combination than with RAIT alone (80%)	[12]
Enveloped virus-like particles (VLPs)	Pancreatic cancer (C57BL/6 tumors)	C57BL/6 tumor-bearing mice	Significant reduction in tumor growth; activation of natural killer cells, lymphocytes and antibodies with no autoimmunity	[79]
Anti-Trop2 hRS7-SN-38 (CL2A-SN-38)	Calu-3 (NSCLC), BxPC-3, Capan-1 (pancreatic adenocarcinoma), and COLO 205 (colonic adenocarcinoma) ; solid tumors	Nude mice xenografts; toxicity assessed in Swiss-Webster Mice and cynomolgus monkeys	Significant antitumor effects at nontoxic doses when compared to nontargeting control antibody-drug conjugates (ADCs)	[69]
Human Fab antibody against the Trop2 extracellular domain	Breast cancer	Nude mice xenografts	Inhibited growth	[70]
antiTrop2 monoclonal antibodies	Endometrium, breast, head and neck, colon-rectum, stomach, lung, ovay, prostate, pancreas, kidney, cervix, and bladder (urothelial) tumors	Nude mice xenografts	Inhibition of tumor growth	[76]
TF12 and IMP288	Prostate cancer	Nude mice xenografts	High and fast accumulation in the tumor, with significant; improvement of survival	[78]
Milatuzumab (antibody-drug conjugate [ADC] of the humanized anti-CD74 antibody)	A-375 (melanoma), HuH-7 and Hep-G2 (hepatoma), Capan-1 (pancreatic), NCI-N87 (gastric), and Raji Burkitt lymphoma	Nude mice xenografts	Increased survival	[79]
bsHexAbs	Lymphoma	SCID mice xenografts	Increased survival; anti lymphoma activity	[56]

#### B. *In-vitro* studies

##### 1. Anti-Trop2 CTL

Human cytotoxic T lymphocytes (CTL) are able to recognize Trop2. Engineered professional antigen-presenting cells (APC) have been made that stimulate the growth and cytotoxicity of specific antitumor CTL in Trop2-expressing lymphomas in murine fibroblast and epithelial cell lines transfected with *Tacstd2*. These Trop2-specific CTL's demonstrate high specific cytotoxic properties against their transfected target, *Tacstd2* [66].

##### 2. Ranpirnase (Rap)

Rap is an amphibian RNase that can act as an antitumor agent and as an immunogen that will not cause any immune response in patients. A Rap fusion protein has been made with the anti-Trop2 antibody hRS7. In lymphoma cell lines, 100% cell killing, *in vitro,* was established at concentrations greater than or equal to 1nM of the specific construct. This fusion protein has potential in inhibiting CLL and Raji Burkitt lymphomas [12].

##### 3. (−)-Epigallocatechin-3-gallate (EGCG)

EGCG is a green tea catechin that can act as an anti-tumorigenic agent, causing suppression of Trop2 expression by affecting the post-transcriptional processing of Trop2 mRNA in SW480 colorectal cells lines. EGCG also affects Trop2 expression at the post-translational level in HCT-116 colorectal cancer cell lines. Treatment with EGCG causes rapid degradation of the Trop2 protein in colorectal cancer cells [47].

##### 4. hRS7

hRS7 is a humanized monoclonal anti-Trop2 antibody that can act as an inhibitor of cancer cell lines (OMMT, UMMT, EEC) and refractory cervical carcinoma [2,67,68]. Numerous hRS7 conjugate drugs also exist [69]. Incubation with hRS7 resulted in a high degree of immune-mediated cell death in certain Trop2 overexpressing ovarian and uterine cell lines. Primary ovarian tumors showing high Trop2 expression, regardless of their serous or clear cell histology, are highly susceptible to hRS7-mediated antibody-dependent cellular cytotoxicity (ADCC) in the presence of effector cells *in vitro.* Although these tumor cells are resistant to multiple standard cytotoxic therapies in clinic studies, they remain highly sensitive to lysis by NK (natural killer) cells when these are engaged by hRS7. The administration of low doses of IL-2 *in vivo* may be a useful adjunct to increase the efficacy of hRS7 cytotoxicity in chemotherapy-resistant ovarian cancer patients. In EEC and refractory cervical cancer, hrS7 treatment also induces ADCC in Trop2 expressing cancer cells. Negligible cytotoxicity against chemotherapy-resistant ovarian cancers was seen in the absence of hRS7 or in the presence of a rituximab control antibody [58].

##### 5. Human Fab antibody against Trop2

The human Fab anti-Trop2 antibody inhibits the proliferation, induces the apoptosis and suspends the migration of MDA-MB-231Trop2-expressing breast cancer cells in a concentration-dependent manner. It is a promising therapeutic agent for breast cancers that express Trop2 [70].

##### 6. Nano drug delivery of apoptosis activator 2

Nano drug delivery of apoptosis activator 2 to human gastric adenocarcinoma (AGS) cell lines with liposome targeted Trop2 antigens is a possible way for smart killing of AGS cells. The apoptosis activator 2-loaded liposomes target cell surface Trop2 antigens in cancer cells and significantly increase apoptosis [71].

##### 7. 5-Aza-2′-deoxycytidine

5-Aza-2′-deoxycytidine treatment on lung cancer cell lines CL1-5, and A549, reverses the hypermethylation of the Trop2 promoter CpG island and elevates both Trop2 mRNA and protein expression in lung cancer cells. This increase in Trop2 expression suppresses lung cancer cell proliferation and colony formation and could, thus, be a therapeutic agent [7].

#### C. *In vivo* studies

##### 1. 131I-IMP-R4-hRS7

hRS7 (an anti-Trop2 monoclonal antibody) labeled with 131I-IMP-R4, has been developed and evaluated in preclinical radioimmunotherapy (RAIT) studies. Nude mice with established (0.3 cm) subcutaneous breast cancer MDA-MB-468 tumors received single administrations of either 131I-IMP-R4-hRS7 or 131I-hRS7 and led to a mean tumor volume 8 weeks post-treatment of 20% compared to 163% for 131I-hRS7 and 280%, respectively, for the control groups. Complete remission was reported in 5 out of 11 mice treated with 131I-IMP-R4-hRS7 [72].

##### 2. 2L-Rap(Q)-hRS7

A novel IgG-based immunotoxin has been made, called 2L-Rap(Q)-hRS7, made of Rap(Q), a mutant Rap with the putative N-glycosylation site removed, and hRS7, an internalizing, humanized antibody against Trop2. This immunotoxin suppresses tumor growth in a prophylactic model of mice with human non-small cell lung cancer. 2L-Rap(Q)-hRS7 could be a potential therapeutic for Trop2 expressing cancers, such as cervical, breast, colon, pancreatic, ovarian, and prostate. In NSCLC xenografts, this immunotoxin suppressed tumor growth, with an increase in the median survival time from 55 to 96 days (P<0.01) [73].

##### 3. 90Y-Hpam4 RAIT (Radioimmunotherapy) with the hrs7-SN-38 conjugate

The humanized antibody 90Y-hPAM4 (90Y-clivatuzumab tetraxetan), when combined with RAIT targets a pancreas cancer antigen, while the conjugate hRS7-SN-38 targets Trop2 on the tumor. This combination leads to a higher survival rate (100%) and greater tumor-free response in animals (90%) than with RAIT alone, which had an 80% survival rate and a 50% tumor free response in animals with tumors of NHL and pancreatic cancer [12].

##### 4. Enveloped virus-Like particles (VLPs)

Immunization with mTrop2 VLPs led to a significant reduction in tumor growth accompanied by a broad activation and tumor infiltration of CD4 (+) and CD8 (+) T cells as well as natural killer and natural killer T cells in pancreatic cancer in mice. VLP immunization generated mTrop2-specific cytotoxic T lymphocytes and antibodies with no measurable induction of autoimmunity. It decreased the population of regulatory T cells and myeloid-derived suppressor cells inside the tumor tissue resulting in decreased levels of immunosuppressive cytokines like interleukin-10 and transforming growth factor-P while promoting the activation of immature macrophages and dendritic cells. VLP immunization with gemcitabine treatment increased the survival of tumor bearing mice [74].

##### 5. Anti-Trop2 hRS7-CL2A-SN-38 antibody-drug conjugate (ADC)

SN-38, the active metabolite of the topoisomerase inhibitor irinotecan, has a derivative, CL2A that has been successfully conjugated to hRS7 to provide significant and specific antitumor effects against a range of human solid tumor types, specifically in the NSCLC cell line Calu-3, the pancreatic cancer cell lines Capan-1 and BxPC-3, and the colon cancer cell line COLO 205. It is well tolerated in monkeys and may work similarly in humans [69,75].

##### 6. Human Fab antibody against the Trop2 extracellular domain

Trop2 Fab inhibited the growth of breast cancer xenografts and reduced expression of anti-apoptotic bcl-2 while elevating the expression of pro-apoptotic Bax [70].

##### 7. Monoclonal antibodies

There is a patent for anti-Trop2 monoclonal antibodies with high affinity that are able to recognize different regions of the Trop2 molecule and can, thus, be used in the treatment and diagnosis of endometrium, breast, head and neck, colon-rectum, stomach, lung, ovary, prostate, pancreas, kidney, cervix, and bladder (urothelial) tumors. Studies of mice injected with human tumors that express Trop2 showed that these monoclonal antibodies can inhibit the growth of tumors [76].

##### 8. TF12 and IMP288

TF12, which is an anti-Trop2 x antihapten bispecific antibody, and ^111^In-IMP288, which is a radiolabeled hapten-peptide, show high and fast accumulation in the tumor, despite TF12's internalizing properties. One cycle of treatment with TF12 and ^177^Lu-IMP288 showed significant improvement of survival compared to treatment with ^177^Lu-IMP288 alone in mouse prostate cancer (90 vs. 67 days, p<0.0001) with no renal or hematological toxicities [77,78].

##### 9. Milatuzumab

Milatuzumab is an antibody-drug conjugate (ADC) of the humanized anti-CD74 antibody. Milatuzumabdoxorubicin was most effective in the lymphoma model in nude mice, whereas in A-375 and Capan-1 solid tumors, only milatuzumab-SN-38 showed increased survival and a therapeutic benefit. Despite much lower surface expression of CD74 than Trop2 or CEACAM6, milatuzumab-SN-38 had similar efficacy in Capan-1 as anti-Trop2-SN-38, but in NCI-N87, anti-CEACAM6 and anti-Trop2 conjugates were superior. CD74 is a suitable target for ADCs in some solid tumor xenografts, with efficacy largely influenced by uniformity of CD74 expression and with SN-38 conjugates providing the best therapeutic responses; SN38 conjugates were preferable in solid cancers, such as melanoma, hepatoma, pancreatic, and gastric, whereas doxorubicin ADC was better in lymphoma [79].

##### 10. bsHexAbs

Trop2 is one of the antibodies used in the construction of bsHexAbs, bispecific hexavalent antibodies, which use the Dock-and-Lock (DNL) method for therapy of treating malignancies [56]. DNL is a method for building bioactive molecules with multifunctional structures [80]. The C_K_-format has advantages over the C_H_3-format as revealed by preclinical results due to improved anti-lymphoma activity *in vivo.* BsHexAbs targets CD20 and CD22 in CLL and Raji Burkitt lymphoma cells and increases chances of survival based on mice xenograft experiments [56].

### Clinical Trial with IMMU-132: Phase I/II Study of IMMU-132 in Patients With Epithelial Cancers

IMMU-132, which targets the Trop2 antigen, is being evaluated as a single agent in previously treated patients with advanced epithelial cancer including ovarian, breast (triple negative), prostate (hormone refractory), lung (non-small cell and small cell), head and neck (squamous cell), esophageal, gastric, colorectal, pancreatic, hepatocellular, renal (clear cell), and bladder cancer. The antibody, RS7, is attached to SN38, which is the active metabolite of irinotecan. This is for Stage IV, metastatic disease for patients who have been treated previously [81].

## CONCLUDING REMARKS

Several antibodies, conjugates, and combination therapies have been developed and implemented in *in vitro* studies and in animal studies, with therapeutic outcomes. Trop2 can be targeted in order to identify its expression level and, thus, the severity of a specific cancer or it can be targeted in order to decrease its expression and, thus, to inhibit tumor progression and decrease tumor size. A human clinical trial is under way for phase I and II treatments of Trop2-expressing epithelial cancers with the antibody conjugate, IMMU-132. Trop2 has a lot of potential as a cancer therapeutic agent. Further, Trop2 expression level may serve as a prognostic biomarker for numerous cancers.

### Future Directions

The Trop2-mediated signaling pathways and transcription factors that regulate its expression are still unclear. An expansion of the mechanisms of Trop2 regulation and miRNA regulation of Trop2 expression along with the targeted genes are warranted for further investigations. Research comparing the direct targeting of Trop2 as opposed to targeting other factors that regulate Trop2 would be helpful. Elucidating the differences of Trop2 expression (over or under expression) in certain cancers and disease stages would be vital to uncovering the exact role of Trop2 in cancer growth and metastasis. The development of novel Trop2 therapies for later stages of cancers as well as therapies that do not induce a toxicity risk to normal Trop2-expressing tissues are vital. Immunotherapy specific for Trop2 can be developed, via specific epitopes as well as immuno-conjugates. Combination therapies have a lot of potential, for instance, the use of agents targeting Trop2 along with conventional chemotherapy, immunotherapy, radioimmunotherapy (RAIT), and chemical inhibitors are warranted investigations in pre-clinical and clinical studies.

## ABBREVIATIONS

ADC: Antibody-drug conjugate
ADCC: Antibody-dependent cell-mediated cytoxicity
AECs: Alveolar epithelial cells
AIRE: Autoimmunity regulator transcription factor
AKT: Protein kinase B
ALCL: Anaplastic large cell lymphoma
AP-1: Activator protein 1
APC: Anitgen-presenting cells
bcl-2: B-cell lymphoma 2
Bmp: Bone morphogenic protein
bsHexAbs: Bispecific hexavalent antibodies
Ca^2+^: Calcium
CaMKII: Calcium/calmodulin-dependent protein kinase II
CBP: CREB-binding protein
Cdc42: Cell division control protein 42
CDK: Cyclin-dependent kinase
CG: Cytosine guanine
CLL: Chronic lymphocytic lymphoma
CPD: Cysteine-poor domain
CpG: Cytosine phosphate guanine
CREB1: Cyclic AMP-responsive-element binding protein
CRD: Cysteine-rich domain
DAG: Deacylglycerol
DNL: Dock-and-lock
E: Embryonic-day
EC: Ectodomain
ECD: Extracellular domain
EEC: Endometrial endometrioid carcinoma
EGF: Epidermal growth factor
EGCG: (−)-Epigallocatechin-3-gallate
EGP/−1: epithelial glycoprotein/−1
EMT: Epithelial to mesenchymal transition
ENK/TL: extranodal nasal type lymphoma
EpCAM: Epithelial cell adhesion molecule
ER: Endoplasmic reticulum
Era: Estrogen receptor a
ERG: ETS-related gene
ERK1/2: Extracellular signal-regulated kinase 1/2
ETS: Erythroblast transformation-specificity
FAK: Focal adhesion kinase
FasL: Fas ligand
FI: Forskolin and IBMX
FIGO: International Federation of Gynecology and Obstetrics
FN: Fibronectin
FOXM1: Forkhead box M1
FOXO3a: Forkhead box O3
GA7331: Gastrointestinal tumor-associated antigen
GDLD: Gelatinous Drop-Like Corneal Dystrophy
Glis2: Glis family zinc finger 2
GRHL-1/3: Grainyhead-like epithelial transactivator-1/3
G0: Resting phase
G1: Growth 1 phase
HER-2: Human epidermal growth factor 2
HNF1A: Hepatocyte nuclear factor 1A
HNSCC: Head and neck squamos cell carcinoma
HOX10A: Homeobox-leucine zipper 10 A protein
IC: Intracellular
ICD: Intracellular domain
IGF-1: Insulin-like growth factor 1
IGF-1R: Insulin-like growth factor 1 receptor
IgG: Immunoglobin G
IKK: IkB kinase
IL-2: Interleukin 2
IN: Intraepithelial neoplasia
IP_3_: Inositol 1,4,5-trisphosphate
LEF1: lymphoid enhancer factor 1
LOH: Loss of heterozygosity
LSCC: Left-sided colon cancer
MAPK: Mitogen-activated protein kinase
MDM2: Mouse double minute 2
MEK: MAPK/ERK kinase
mTrop2: Murine Trop2
MMP: Matrix metalloproteinase
M1S1: Membrane component chromosome 1 surface marker 1
NF-κB: Nuclear factor-κB
NHL: Non-Hodgkin's lymphoma
NK: Natural killer
NSCLC: Non-small cell lung carcinoma
OMMT: Ovarian mixed mullerian tumor
PBL: Peripheral blood lymphocytes
pPdpn: Podoplanin
ERK: phosphorylated ERK
PH: pleckstrinhomology
PIP_2_: Phosphatidylinositol 4,5-bisphosphate
PKC: protein kinase C
PLC: Phospholipase C
PS-1/-2: Presenilin-1/-2
P13K: Phosphoinositide 3-kinase
qRT-PCR: Quantitative reverse transcriptase polymerase chain reaction
RACK1: Receptor for activated protein kinase C
RAIT: Radioimmunotherapy
Rap: Ranpirnase
Rb: Retinoblastoma protein
RhoaA: Ras homolog gene family, member Aa
RIP: Regulated intramembrane proteolysis
ROS: Reactive oxygen species
RSCC: Right-sided colon cancer
S phase: Synthesis phase
SCID: Severe combined immunodeficiency
S294/344/425: Serine 294/344/425
SP1: Specificity protein 1
SPI1: Spleen focus forming virus proviral integration oncogene
Src: Tyrosine-protein kinase
STAT1: Signal transducer and activator of transcription 1
STAT3: Signal transducer and activator of transcription 3
TACE: Tumor necrosis factor a converting enzyme
TCF-1: T-cell factor 1
TGF-β: Transforming growth factor P
TY: Thyroglobin type-1
TM: Transmembrane helix
TP63/53L: Tumor protein 63/53L
UMMT: Uterine mixed mullerian tumor
UMSCC: University of Michigan squamos cell carcinoma
USPC: Uterine serous papillary carcinoma
Vegf: Vascular endothelial growth factor
VLPs: Enveloped virus-like particles
WHO: World Health Organization
WT1: Wilms' tumor suppressor gene
